# Novel Food and Beverages: Production and Characterization: 2nd Edition

**DOI:** 10.3390/foods14132349

**Published:** 2025-07-02

**Authors:** Víctor M. Perez-Puyana, Alberto Romero, Mercedes Jiménez-Rosado

**Affiliations:** 1 Departamento de Ingeniería y Ciencia de los Materiales y del Transporte, Universidad de Sevilla, 41092 Sevilla, Spain; 2 Departamento de Ingeniería Química, Universidad de Sevilla, 41012 Sevilla, Spain; alromero@us.es; 3 Chemical, Environmental and Bioprocess Engineering Group, I4 Institute, University of León, 24071 León, Spain; mjimr@unileon.es

## 1. Introduction

In today’s food landscape, the development of novel products continues to be a critical response to the growing demand for healthier, more sustainable, and technologically advanced food and beverage options [1,2]. The increasing complexity of consumer expectations, combined with resource limitations and market competition, reinforces the need for constant product optimization and innovation [3,4].

Current efforts from both academia and industry are focused on enhancing the nutritional profile of products, exploring alternative raw materials, and improving processing techniques to achieve better performance and shelf life [5]. In parallel, research is also targeting the integration of bioactive compounds, evaluation of functionality, and assessment of consumer acceptance through multidisciplinary approaches [6,7].

This Editorial corresponds to the Special Issue *“Novel Food and Beverages: Production and Characterization: 2nd Edition”*, which builds upon the foundation laid by the previous edition while broadening its scope to include emerging challenges and innovative solutions. Both Special Issues are focused on novel food sources for the development of innovative food and beverages, paying attention to several advanced technologies for the analysis and quality assessment of these new products, such as chromatography, spectroscopy, or ultrasound, among others. This issue captures a rich array of contributions that reflect ongoing progress in the design, enrichment, and analysis of novel food matrices. Emphasis is placed on the valorization of by-products, the integration of health-promoting components, the study of rheological and sensory properties, and the advancement of sustainable approaches in formulation. Collectively, these works contribute to a deeper understanding of how innovation in food production can meet the dual demands of nutritional quality and environmental responsibility. In other words, this Special Issue contributes to the 2030 Sustainable Development Goals (SDGs). In particular, it connects SDG 2 with SDG 12, since it combines the search for novel food sources (ensuring sustainable production patterns) with health (improving nutrition).

## 2. Overview of Published Articles

Seven manuscripts were submitted for the Special Issue. After a rigorous review process, five papers were accepted for publication in the Special Issue, each offering a unique perspective on the development, enrichment, and characterization of novel food and beverage products with functional or sustainable attributes.

One study by Jabeen et al. focused on the formulation of functional beverages based on germinated brown rice enriched with bioactive compounds like γ-aminobutyric acid. Their results revealed significant improvements in nutritional properties, including elevated levels of γ-oryzanol, total phenolics, niacin, and γ-aminobutyric acid. These beverages presented favorable antioxidant activity and consumer acceptance, positioning them as promising alternatives for health-conscious individuals.

In the domain of functional meat alternatives, Cardoso et al. developed an innovative mackerel, quinoa, and seaweed-based hamburger aimed at supporting cognitive health. By employing different cooking methods (steaming, roasting, and grilling), they demonstrated that the final products retained high levels of essential nutrients such as docosahexaenoic acid, iodine, and selenium, as well as beneficial n-3 polyunsaturated fatty acids, thereby providing a compelling option for brain health through dietary intervention.

The valorization of floral by-products was explored by Cerdá-Bernad et al., who formulated model beverages using floral saffron residues. Their work examined the impact of thermal processing and thickeners on polyphenol composition, rheological properties, and the inhibition of digestive enzymes. Notably, only heat treatment formulations showed α-amylase inhibitory activity, suggesting a critical interaction between matrix components and processing techniques for enhancing functionality.

Multisona et al. turned their attention to baked goods, enriching cookies with varying concentrations of *Clitoria ternatea* (CT, butterfly pea flower) petals. Their findings identified the 6% inclusion level as optimal, improving fiber content, antioxidant activity, and sensory attributes. Despite some increase in peroxide values over time, the fortified cookies retained good visual and microbial quality, demonstrating the viability of this botanical ingredient in health-promoting bakery products.

Lastly, Marques et al. conducted a comprehensive characterization of Iberian wine vinegars, analyzing their amino acid composition and the presence of tryptophan-derived bioactive molecules. The study revealed the nutritional and functional potential of various regional vinegars (white, red, port, and balsamic) highlighting their content of compounds such as arginine, alanine, and threonine, all linked to cardiovascular and immune system health.

Together, these contributions reflect the diversity and innovation at the heart of this Special Issue. They not only exemplify the scientific progress in developing functional foods and beverages, but also underline the increasing importance of sustainability, bioactivity, and consumer-centered design in contemporary food research.

## 3. Conclusions and Future Perspectives

The increase in the global human population (estimations at around 10 billion people by 2050) and longer life expectancy makes the search for healthy diets one of the key remaining challenges of the 2030 SDGs. Therefore, food production needs to be optimized to fulfill the future demand. In light of this, scientists are pursuing efforts to ensure sustainable food production. Therefore, research in this field is essential to achieve this goal, paying special attention to the following approaches:

- The search for unexplored plant and animal sources of new ingredients, considering the possible emergence of new pathogens and foodborne diseases;
- Improve processing technologies to make them more cost-affordable and environmentally friendly;
- Incorporate new supplements to improve the nutritional quality of the diet;
- Life cycle analysis of food production, paying attention to the new consumer demands.

The studies gathered in this Special Issue illustrate key advances aligned with these objectives. From the development of functional beverages and bakery products to the revalorization of by-products and the examination of traditional foods with renewed scientific insight, each contribution provides a foundation for future work that bridges science, health, and sustainability in food innovation.

## Data Availability

Not applicable.
